# ExpI and PhzI Are Descendants of the Long Lost Cognate Signal Synthase for SdiA

**DOI:** 10.1371/journal.pone.0047720

**Published:** 2012-10-17

**Authors:** Anice Sabag-Daigle, Brian M. M. Ahmer

**Affiliations:** 1 Department of Microbiology, The Ohio State University, Columbus, Ohio, United States of America; 2 Center for Microbial Interface Biology, The Ohio State University, Columbus, Ohio, United States of America; Indian Institute of Science, India

## Abstract

SdiA of *E. coli* and *Salmonella* is a LuxR homolog that detects *N*-acyl homoserine lactones (AHLs). Most LuxR homologs function together with a cognate AHL synthase (a LuxI homolog), but SdiA does not. Instead, SdiA detects AHLs produced by other bacterial species. In this report, we performed a phylogenetic analysis of SdiA. The results suggest that one branch of the *Enterobacteriaceae* obtained a *rhlR*/*rhlI* pair by horizontal transfer. The *Erwinia* and *Pantoea* branches still contain the complete pair where it is known as *expR*/*expI* and *phzR*/*phzI*, respectively. A deletion event removed the *luxI* homolog from the remainder of the group, leaving just the *luxR* homolog known as *sdiA*. Thus ExpR and PhzR are SdiA orthologs and ExpI and PhzI are descendants of the long lost cognate signal synthase of SdiA.

## Introduction

Quorum sensing in the *Proteobacteria* often utilizes LuxI and LuxR pairs in which the LuxI homolog synthesizes an N-acylhomoserine lactone (AHL) that can enter and exit the cell using diffusion or efflux pumps, depending on the AHL type [1]–[4]. If the bacterial population is at high population density in a confined space (if it has reached a quorum), then AHL will accumulate and will be detected by the LuxR homolog, which contains an AHL binding domain and a DNA binding domain. Gene expression is then regulated accordingly by the LuxR homolog (reviewed in [5]–[7]). Many Proteobacteria encode LuxR homologs that are not paired with a cognate AHL synthase. These have been termed solo or orphan LuxRs [8], [9]. *E. coli* and *Salmonella* encode a single *luxR* homolog named *sdiA* but do not encode a *luxI* homolog or any other type of AHL synthase (reviewed in [10]). It has been shown that *E. coli* and *Salmonella* detect the AHLs produced by other species of bacteria in an *sdiA*-dependent manner [11]–[15]. Previous reports have shown that the SdiA orthologs are most closely related to RhlR from *Pseudomonas aeruginosa*. Therefore, it has been proposed that SdiA arose from a horizontal gene transfer event of *rhlR* from a pseudomonad [16], [17]. However, it has never been determined whether the *rhlR* homolog was obtained alone, or paired with a *luxI* homolog that was later lost.

Individual LuxI homologs synthesize distinct AHLs. The AHLs can vary in chain length and saturation, and in modifications at the third carbon, where they may or may not have a keto group or a hydroxy group. The name of the AHL can be abbreviated by describing the chain length and the type of modification. For example, LuxI of *Vibrio fischeri* synthesizes primarily oxoC6 (N-(3-oxo-hexanoyl)- L-homoserine lactone), which has a 6-carbon tail with a keto modification at the third carbon. Accordingly, the *V. fischeri* LuxR protein binds oxoC6 [18]–[20]. Thus, the *V. fischeri* LuxI and LuxR form a signal generating and detecting pair. The SdiA protein of *Salmonella* binds a wide range of AHLs (C4 to oxoC12) but detects oxoC6 and oxoC8 with the highest sensitivity [11], [21].

## Results and Discussion

**Figure 1:**
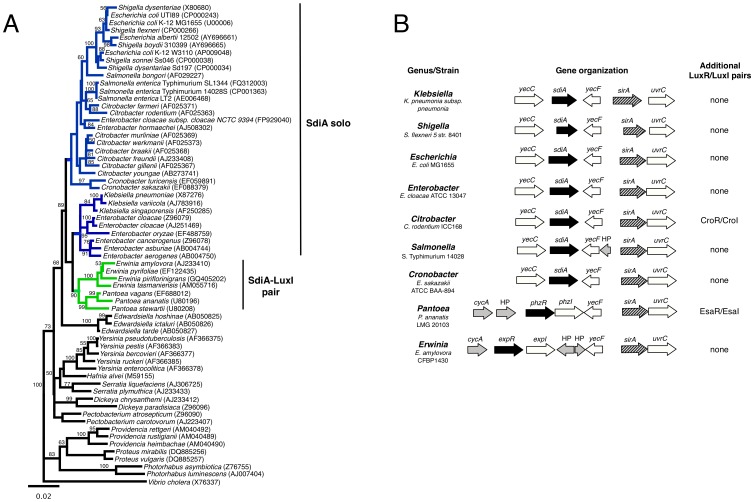
Genomic organization and distribution of *sdiA* in the *Enterobacteriaceae*. a) Distribution of *sdiA* on a phylogenetic tree based on 16S rDNA sequences. Only bootstrap values of ≥50 are displayed. Blue lines indicate species that contain a solo *sdiA*. Green lines indicate species that contain *sdiA* and an adjacent *luxI* homolog. A maximum likelihood tree gave similar results (not shown). b) Map of the *sdiA* region in representative organisms that encode SdiA orthologs. Any additional LuxR/LuxI pairs in those organisms are also listed. Genes depicted in white are conserved in all genera and genes in gray are not conserved. *sdiA* is represented in black and *sirA* in gray hatched lines.

**Figure 2:**
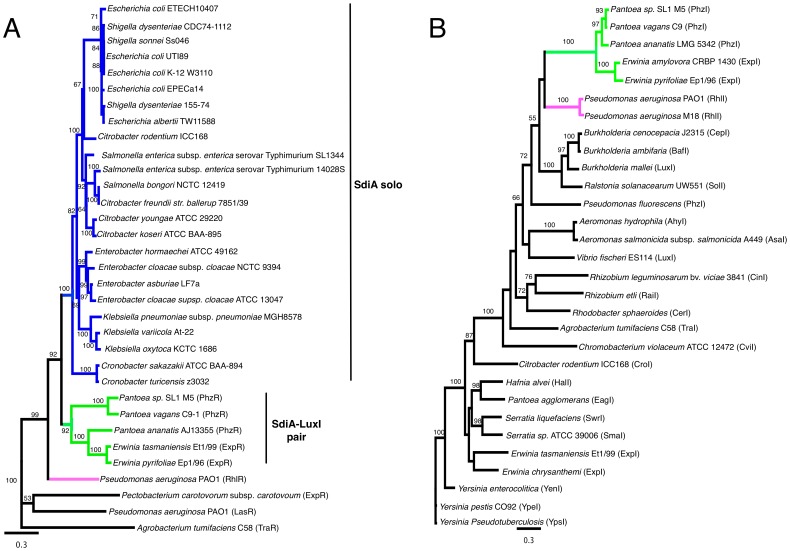
Phylogeny of LuxR and LuxI homologs. a) A phylogenetic tree of LuxR homologs. Only bootstrap values of ≥50 are displayed. A maximum likelihood tree gave similar results (not shown). Blue lines indicate species that contain a solo *sdiA*. Green lines indicate species that contain *sdiA* and an adjacent *luxI* homolog. Pink lines indicate species that contain *rhlR.* b) A phylogenetic tree of LuxI protein sequences. Only bootstrap values of ≥50 are displayed. A maximum likelihood tree gave similar results (not shown). Green lines indicate species that contain *sdiA* and an adjacent *luxI* homolog. Pink lines indicate species that contain *rhlR.*

A survey of the genomic organization of *sdiA* in *S.* Typhimurium, *E. coli*, *Enterobacter, Citrobacter*, and *Klebsiella* showed that *sdiA* is always present upstream of *sirA* (*Salmonella* invasion regulator), the response regulator of a two-component regulatory system, and downstream of the uncharacterized *yecC* gene (Fig. 1B). SirA orthologs have been given a variety of different names, including *uvrY* in *E. coli*, *gacA* in *Pseudomonas*, *letA* in *Legionella*, *expA* in *Erwinia*, and *varA* in *Vibrio*. We utilized the genomic context of *sdiA* upstream of sirA to classify LuxR homologs as SdiA orthologs. With this criteria, we searched the annotation data of the 3911 draft and completed genomes (as of June 2012) deposited at the Pathosystems Resource Integration Center (PATRIC) for SdiA protein sequences (FIG00004070) [22]. There are 360 members of the SdiA protein family all of which are found within the *Enterobacteriaceae* family (Figure 1A). Surprisingly, ExpR and PhzR from *Erwinia* and *Pantoea* are classified as members of the SdiA family and they are located near *sirA* (Fig. 1B). However, these should not be confused with ExpR from *Pectobacterium* or the EsaR in *Pantoea*, which are not *sdiA* orthologs and are not located near *sirA*. A phylogenetic tree of SdiA protein sequences shows that ExpR and PhzR are more closely related to SdiA than to other LuxR homologs (Figure 2A). Based on their homology and genomic context near *sirA* we propose that ExpR of *Erwinia* and PhzR of *Pantoea* are SdiA orthologs.

Interestingly, ExpI and PhzI are most closely related to RhlI (Figure 2B). Therefore, we propose that a complete *rhlR/rhlI* pair was acquired horizontally with the deletion of *rhlI* after divergence of *Escherichia*, *Salmonella*, and close relatives, from *Pantoea* and *Erwinia*. ExpI and PhzI produce oxoC6 [23], [24], an AHL detected with high sensitivity by SdiA [9], which is consistent with ExpI and PhzI representing descendants of the ancient LuxI protein paired with SdiA.

While the production of oxoC6 by ExpI and PhzI, and the detection of oxoC6 by ExpR, PhzR, and SdiA are all consistent, the earliest event, the acquisition of a RhlR/RhlI pair, has an inconsistency. RhlI synthesizes C4 and RhlR is thought to be primarily a C4 receptor [25], [26]. Therefore, while unlikely, it is possible that RhlR and RhlI are not the direct ancestors of the Exp, Phz and SdiA systems. If RhlR and RhlI are indeed the ancestors, then there are several possible explanations for the signal discrepancy. The most likely hypothesis is that the signal(s) generated and detected have simply diverged. More complicated explanations include the possibility that differences in the host organisms, such as the availability of fatty acids or acyl carrier proteins (ACP), cause different signals to be produced by the LuxI homolog or that differences in efflux pumps or membrane permeability change the AHLs available for detection by the LuxR homolog, thus altering the apparent specificity of the LuxR homolog [3], [4], [27]–[30]. For instance, it is known that RhlR can detect oxoC6, at least when expressed in *E. coli* and *Salmonella*, and purified RhlI can synthesize C6 when provided with hexanoyl-ACP and SAM [28], [31].

In conclusion, we propose that ExpR and PhzR are SdiA orthologs, and that ExpI and PhzI are descendants of the missing cognate AHL synthase for SdiA of *Escherichia*, *Salmonella* and other relatives where SdiA is a solo LuxR homolog. Further studies are needed to determine if these systems are descended from RhlR/RhlI, and whether there are indeed signal differences between the systems, and if so, the causes of those differences.

## Materials and Methods

### 

#### 16S rDNA trees

The 16S rDNA sequences from 64 species were obtained and aligned using The Ribosomal Database Project (RDP) [32]. The alignment was downloaded in phylip format and imported into Geneious [33]. Geneious tree builder was used to generate a neighbor-joining tree with 100 bootstrap replicates [33]. An alternative tree using a second method, maximum likelihood, was generated using PhyML in Geneious with the same 16S rDNA phylip alignment file [34]. This method used a Jukes-Cantor substitution method with 100 bootstrap replicates.

#### SdiA and LuxI phylogeny trees

The sequences of representative SdiA and LuxI proteins were collected from GenBank. These protein sequences were aligned in Geneious using MUSCLE with 8 iterations [33]. The alignment was then used to generate a neighbor-joining tree with 100 bootstrap replicates. An alternative tree using a second method, maximum likelihood, was generated using PhyML in Geneious with the same MUSCLE alignment file [34]. This method used a Jukes-Cantor substitution method with 100 bootstrap replicates.
